# The Crisis Once More

**Published:** 1921-05-21

**Authors:** 


### THE CRISIS ONCE MORE.

ALTHOUGH the report of Lord Cave's Committee is expected at the end of the month, and it may contain some practical method of extrication from the grave financial position in which hospitals find themselves, here and there are to be seen signs of individual hospitals throwing up the sponge. Again it is announced that the London Hospital is to close; or rather, to close 200 beds and to cut down the out-patients to 35,000. King's College Hospital, if anything worse off, is forced to reduce the available number of beds by 400. And while these regrettable decisions are being made, unemployment, bringing under-nourishment in its train, which must accentuate the need for hospital treatment, is becoming more widespread every day.

What are the solutions? The Earl of Northampton, chairman of the Great Northern Central Hospital, appeals for a lump sum from National Health Insurance accumulations as a payment for past services to insured patients. He and Lord Cave approve the action of the National Deposit Friendly Society in allocating one-third of its surplus to hospital and nursing organisations, and the latter expresses the hope that other societies, great and small, will follow suit. From another source comes the spendthrift suggestion that the banks should be more liberal in their loans, and there is a move to obtain Parliamentary sanction for hospitals to dip into their trust funds to provide the means of maintenance. These doubtful financial expedients, in our opinion, may help for a brief time, but will eventually hinder.

A far more practical suggestion, although it will help only some of the hospitals, is that the Government should recognise its liabilities in respect of payment for the treatment of service men during the war. The four shillings a day, increased later by a few pence at the instance of the not then so authoritative British Hospitals Association, was insufficient to cover the cost of treatment. An immediate payment of 50 per cent. over all grants made would ease the financial stress of a large number of voluntary hospitals and would leave the central funds with a much freer hand to deal with the remainder. In the meantime we must spare no effort to secure future financial support by such means as the Pageant we have proposed, and thus educate the public to secure to themselves the continued advantages of one of their most precious heritages.

